# Supplementary material for: Over half of western United States' most abundant tree species in decline
Source: Nat Commun. 2021 Jan 19;12:451. doi: 10.1038/s41467-020-20678-z (PMC7815881; doi:10.1038/s41467-020-20678-z)
Supplement: Supplementary file 2 — Reporting Summary [file 41467_2020_20678_MOESM2_ESM.pdf]

nature research

Corresponding author(s): Hunter Stanke

Last updated by author(s): Nov 13, 2020

Reporting Summary

Nature Research wishes to improve the reproducibility of the work that we publish. This form provides structure for consistency and transparency in reporting. For further information on Nature Research policies, see our Editorial Policies and the Editorial Policy Checklist.

Statistics

For all statistical analyses, confirm that the following items are present in the figure legend, table legend, main text, or Methods section.

n/a	Confirmed	
☐	☒	The exact sample size (*n*) for each experimental group/condition, given as a discrete number and unit of measurement
☐	☒	A statement on whether measurements were taken from distinct samples or whether the same sample was measured repeatedly
☒	☐	The statistical test(s) used AND whether they are one- or two-sided<br>*Only common tests should be described solely by name; describe more complex techniques in the Methods section.*
☐	☒	A description of all covariates tested
☐	☒	A description of any assumptions or corrections, such as tests of normality and adjustment for multiple comparisons
☐	☒	A full description of the statistical parameters including central tendency (e.g. means) or other basic estimates (e.g. regression coefficient) AND variation (e.g. standard deviation) or associated estimates of uncertainty (e.g. confidence intervals)
☒	☐	For null hypothesis testing, the test statistic (e.g. *F*, *t*, *r*) with confidence intervals, effect sizes, degrees of freedom and *P* value noted<br>*Give P values as exact values whenever suitable.*
☐	☒	For Bayesian analysis, information on the choice of priors and Markov chain Monte Carlo settings
☐	☒	For hierarchical and complex designs, identification of the appropriate level for tests and full reporting of outcomes
☐	☒	Estimates of effect sizes (e.g. Cohen's *d*, Pearson's *r*), indicating how they were calculated

Our web collection on statistics for biologists contains articles on many of the points above.

Software and code

Policy information about availability of computer code

Data collection	We used the open-source R package, rFIA (0.2.3), to acquire all USDA Forest Inventory and Analysis data used in this study.
Data analysis	All statistical analysis was conducted in Program R (4.0.0). As part of this work, we have implemented the methods presented herein in the rFIA R package (available in version 0.2.3). Hence all code is open source, and our method can be easily extended to other populations of interest and/or study regions using the USDA Forest Inventory and Analysis database. We use custom R code to process our results, and all such code have been made publicly available in an online GitHub repository (https://github.com/hunter-stanke/Code-repository---NCOMMS-20-20430).

For manuscripts utilizing custom algorithms or software that are central to the research but not yet described in published literature, software must be made available to editors and reviewers. We strongly encourage code deposition in a community repository (e.g. GitHub). See the Nature Research guidelines for submitting code & software for further information.

Data

Policy information about availability of data

All manuscripts must include a data availability statement. This statement should provide the following information, where applicable:
- Accession codes, unique identifiers, or web links for publicly available datasets
- A list of figures that have associated raw data
- A description of any restrictions on data availability

All forest inventory data used herein are publicly available via the USDA Forest Service Forest Inventory and Analysis data repository (https://apps.fs.usda.gov/fia datamart/CSV/datamart_csv.html).

Field-specific reporting

Please select the one below that is the best fit for your research. If you are not sure, read the appropriate sections before making your selection.

☐ Life sciences ☐ Behavioural & social sciences ☒ Ecological, evolutionary & environmental sciences

For a reference copy of the document with all sections, see nature.com/documents/nr-reporting-summary-flat.pdf

Ecological, evolutionary & environmental sciences study design

All studies must disclose on these points even when the disclosure is negative.

Study description	Observational study drawing on remeasured USDA Forest Inventory and Analysis plots to quantify temporal changes in relative live tree density of the most abundant tree species in the western United States, map intra-specific variation in relative density shifts, and determine the relative importance of various forest disturbance agents and long-term climate patterns in driving species population performance.
Research sample	Repeated observations of approximately 24,000 USDA Forest Inventory and Analysis (FIA) field plots distributed across 10 states in the western United States (i.e., WA, OR, CA, NV, AR, NM, CO, UT, ID, MT; WY excluded due to a lack of data). We selected the eight most abundant tree species in the region for analysis (i.e., Pseudotsuga menziesii; Pinus contorta; Abies lasiocarpa; Pinus ponderosa; Pinus edulis; Populus tremuloides; Picea engelmannii; and Juniperus osteosperma). All data are publicly available at: https://apps.fs.usda.gov/fia/datamart/.
Sampling strategy	FIA field plot locations are distributed at a rate of approximately 1 plot per 2400 hectares across the continental US to produce a spatially unbiased sample of US forests. Measurements of individual trees are taken on these FIA field plots. Specifically, trees greater than 12.7cm diameter at breast height (d.b.h.) are measured on a cluster of four 168 sq. meter subplots. Trees greater than 2.5cm and less than 12.7cm d.b.h. are measured on one of four microplots (13.5 sq. meter) nested within subplots. In the states of Oregon and Washington, rare events such as very large trees are measured on one of four macroplots (1012 sq. meter) that encompass each subplot. All species assessed herein were observed on over 1000 of such FIA ground plots distributed across their range's in the western US. Species were selected for inclusion in this study by their estimated total abundance (i.e., number of stems) within the region. Specifically, we chose to include the eight most abundant species in the western US in this study. The sample size of this study (i.e., number of plots included) was determined based on data availability in the FIA database. Such samples are sufficiently large for use in the US national forest inventory, and hence should be considered of sufficient size for use in this study.
Data collection	Data were collected by USDA FIA field personnel (hundreds of individuals employed by the United States Forest Service) as part of the United State's national forest inventory. Individual tree diameters were measured with tree calipers and diameter tapes, tree density (i.e., the density represented by an individual tree) is determined from the "fixed area" response design described above.
Timing and spatial scale	All data were collected between 2001 and 2018, with individual field plots being remeasured approximately every 10 years. Each field plot was remeasured once during the remeasurement interval. The study region spans 10 states (approximately 85 million hectares), and field plots were distributed systematically across the region to produce a spatially unbiased sample of forest condition and change in the region. Field plots are distributed at a rate of approximately 1 plot per 2400 hectares.
Data exclusions	For brevity, we limit our analysis to consider the eight most abundant tree species in the western US (representing 61.6% of all live trees in the study region). We exclude the state of Wyoming due to a lack of remeasurement data.
Reproducibility	We have implemented the methods presented herein in the open-source R package, rFIA (available in version 0.2.3). All data used in this analysis are publicly available. Hence our results can be readily reproduced. We have successfully reproduced/replicated this analysis on three independent occasions (i.e., different individuals using different computers/operating systems, all attempts were successful). Furthermore, our methods can be easily extended to other populations of interest and/or spatial extents using the Forest Inventory and Analysis Database. All custom code, along with instructions for use, have been made publicly available in an online GitHub repository (https://github.com/hunter-stanke/Code-repository---NCOMMS-20-20430).
Randomization	FIA field plot locations are psuedo-randomly distributed across the continental US using a hexagonal sampling frame. That is, the land basis of the United States of divided into a large number of independent populations (i.e., spatial hexagons approximately 6000 acres in area). One field plot has been established in each of these populations (hexagons), however the exact location of the field plot within the population is determined randomly. This sampling scheme produces a randomized, yet spatially unbiased sample of the forest land basis across the United States.
Blinding	Blinding was not used in this study as all data were either modeled from automated sampling method (i.e., in the case of climate data), or previously collected as part of a national forest inventory (i.e., in the case of FIA data).

Did the study involve field work? ☐ Yes ☒ No

Reporting for specific materials, systems and methods

We require information from authors about some types of materials, experimental systems and methods used in many studies. Here, indicate whether each material, system or method listed is relevant to your study. If you are not sure if a list item applies to your research, read the appropriate section before selecting a response.

Materials & experimental systems

n/a	Involved in the study
☒	☐ Antibodies
☒	☐ Eukaryotic cell lines
☒	☐ Palaeontology and archaeology
☒	☐ Animals and other organisms
☒	☐ Human research participants
☒	☐ Clinical data
☒	☐ Dual use research of concern

Methods

n/a	Involved in the study
☒	☐ ChIP-seq
☒	☐ Flow cytometry
☒	☐ MRI-based neuroimaging
